# RiboTag Analysis of Actively Translated mRNAs in Sertoli and Leydig Cells *In Vivo*

**DOI:** 10.1371/journal.pone.0066179

**Published:** 2013-06-11

**Authors:** Elisenda Sanz, Ryan Evanoff, Albert Quintana, Elizabeth Evans, Jeremy A. Miller, Chemyong Ko, Paul S. Amieux, Michael D. Griswold, G. Stanley McKnight

**Affiliations:** 1 Department of Pharmacology, University of Washington, Seattle, Washington, United States of America; 2 School of Molecular Biosciences, Center for Reproductive Biology, Washington State University, Pullman, Washington, United States of America; 3 Department of Biochemistry and Howard Hughes Medical institute, University of Washington, Seattle, Washington, United States of America; 4 Allen Institute for Brain Science, Seattle, Washington, United States of America; 5 Department of Comparative Biosciences, College of Veterinary Medicine, University of Illinois at Urbana-Champaign, Urbana, Illinois, United States of America; University Hospital of Münster, Germany

## Abstract

Male spermatogenesis is a complex biological process that is regulated by hormonal signals from the hypothalamus (GnRH), the pituitary gonadotropins (LH and FSH) and the testis (androgens, inhibin). The two key somatic cell types of the testis, Leydig and Sertoli cells, respond to gonadotropins and androgens and regulate the development and maturation of fertilization competent spermatozoa. Although progress has been made in the identification of specific transcripts that are translated in Sertoli and Leydig cells and their response to hormones, efforts to expand these studies have been restricted by technical hurdles. In order to address this problem we have applied an *in vivo* ribosome tagging strategy (RiboTag) that allows a detailed and physiologically relevant characterization of the “translatome” (polysome-associated mRNAs) of Leydig or Sertoli cells *in vivo*. Our analysis identified all previously characterized Leydig and Sertoli cell-specific markers and identified in a comprehensive manner novel markers of Leydig and Sertoli cells; the translational response of these two cell types to gonadotropins or testosterone was also investigated. Modulation of a small subset of Sertoli cell genes occurred after FSH and testosterone stimulation. However, Leydig cells responded robustly to gonadotropin deprivation and LH restoration with acute changes in polysome-associated mRNAs. These studies identified the transcription factors that are induced by LH stimulation, uncovered novel potential regulators of LH signaling and steroidogenesis, and demonstrate the effects of LH on the translational machinery *in vivo* in the Leydig cell.

## Introduction

In mammals, male reproductive capacity is maintained via a complex network of positive and negative feedback loops that act anatomically at the level of the hypothalamus, the pituitary and the gonad (referred to as the HPG axis) [1], [2]. Pulsatile release of Gonadotropin-releasing hormone (GnRH) from neurons located in the preoptic area of the hypothalamus into the hypophysial portal circulation acts on the gonadotropes of the anterior pituitary to cause release of the gonadotropins LH and FSH [3], [4]. LH and FSH act on the somatic Leydig and Sertoli cells of the testis, respectively, to stimulate steroidogenesis and support spermatogenesis, while factors such as the inhibins produced by Sertoli cells and androgens produced by the Leydig cells feedback negatively at the level of the hypothalamus and pituitary to reduce GnRH, LH and FSH levels [5], [6], [7].

Leydig and Sertoli cells are primary responders to circulating gonadotropin hormones and support the development of germ cells. Failure of the somatic cells of the testis to respond appropriately to hormonal cues within the HPG axis or to create the appropriate local spermatogonial stem cell niche can result in male infertility [5], [8], [9]. Pharmacological disruption of Leydig or Sertoli cell function also represents a potential avenue for the development male contraceptives [10].

Although significant progress has been made toward understanding testicular function and global gene expression changes in testis using a combination of surgical, pharmacological or genetic manipulations and genome-scale analysis [5], [11], [12], [13], determining cell type-specific gene expression changes *in vivo* in the testis has been difficult due to lack of appropriate tools. Previous strategies to identify cell-type-specific gene expression in testis have relied on either partially purified cell populations or the use of genetic models such as the hypogonadal mouse (*HPG*), where testis development can be initiated *de novo* by administration of GnRH, gonadotropins or testosterone (T) [11], [13], [14], [15], [16], [17], [18], [19]. However, a cell-specific characterization of the transcriptional dynamics of testicular somatic cells *in vivo* in a physiologically relevant context has yet to be achieved. In the present study, we took advantage of the recently developed RiboTag mouse line [20] to epitope-tag ribosomes from either Leydig or Sertoli cells and isolate cell-specific mRNAs that are actively being translated in the adult mouse *in vivo*. This approach allowed us to identify novel mRNAs expressed in these somatic cell types and provide a detailed characterization of the effect of gonadotropins and testosterone on translatome dynamics in Leydig and Sertoli cells *in vivo*.

## Materials and Methods

### Ethics Statement

All mouse procedures were approved under protocol 2022-01, titled “Regulation of cAMP-Dependent Protein Kinase Genes, by our Institutional Animal Care and Use Committee (IACUC) at the University of Washington, which operates under approval number A3464-01 from the Association for Assessment and Accreditation of Laboratory Animal Care (AAALAC).

### Animal Maintenance and Treatments

Mice were housed in a temperature and humidity controlled facility with a 12-h light/dark cycle. Leydig cell-specific (Cyp17iCre: RiboTag) and Sertoli cell-specific (AMH-Cre: RiboTag) RiboTag mice were obtained by crossing RiboTag homozygous mice [20] with Cyp17iCre [21] or AMH-Cre mice [22]. Cyp17iCre mice were obtained from Dr. CheMyong Ko and AMH-Cre mice were provided by Dr. Robert E. Braun. For *in vivo* LH treatment experiments, mice were injected subcutaneously with 300 ug of the GnRH antagonist acyline (a generous gift of Dr. John K. Amory) every 24 h for 4 days before a single intraperitoneal injection of 2 units of purified human LH (Scripps laboratories). After treatments, mice were sacrificed by CO_2_ asphyxiation or a single Beuthanasia-D injection.

### Immunoprecipitation Assays

After treatments, testes were homogenized and immunoprecipitation was performed as described previously [20] with minor modifications. Briefly, 10 ul of anti-HA antibody (Covance) were coupled to 200 ul of beads in citrate-phosphate buffer pH 5.0 and the antibody-bead complex was added to the cleared homogenates and incubated overnight at 4°C. After incubation, beads were washed in high salt buffer 3 times for 5 min, resuspended in RLT buffer (with beta-mercaptoethanol; Qiagen) and stored at −80°C until RNA extraction.

### LH, FSH and Testosterone Serum Determination

After treatments, blood was obtained by cardiac puncture and allowed to clot in Microtainer serum separator tubes (Becton-Dickinson) for 1 h at RT. Serum was recovered by centrifugation and stored at −80°C for later analysis. LH and FSH serum levels were determined by RIA at the University of Virginia Center for Research in Reproduction Ligand Assay and Analysis Core, and testosterone levels were determined using the Testosterone EIA kit (Cayman Chemical).

### RNA Extraction

RNA from the inputs (50 ul) and the pellets was obtained using the RNeasy mini kit (Qiagen) according to manufacturer's directions. Total RNA was quantified using a NanoDrop 1000 spectrophotometer (Thermo Scientific) and its quality was assessed using an Agilent 2100 Bioanalyzer with the RNA 6000 Nano kit (Agilent Technologies).

### Microarray Analysis

100 ng of RNA was amplified and labeled using NuGen Ovation labeling kit and hybridized to Affymetrix GeneChip Mouse Gene 1.0 ST Arrays. Array output was normalized using the RMA algorithm, and data analysis was conducted using GeneSpring (Version 11.0.2; Agilent Technologies). Genes were considered to be regulated if they 1) had a raw score of greater than 50 in at least one sample, 2) were determined to be significantly different versus controls by ANOVA followed by Contrast analysis (p<0.05) and 3) showed a 2-fold (for IP versus input analysis) or 1.5-fold (for IP analysis) or greater increase or decrease versus controls. All samples in each experiment were included in the statistical analysis. The Affymetrix Raw.CEL files have been deposited with the National Center for Biotechnology Information Gene Expression Omnibus (Accession number: GSE45799; http://www.ncbi.nlm.nih.gov/geo/query/acc.cgi?acc=GSE45799). Gene ontology analysis was performed using the Web-based Gene Set Enrichment Analysis Toolkit (WebGestalt) V2. http://bioinfo.vanderbilt.edu/webgestalt/.

### Hierarchical Clustering Analysis

Data from all Affymetrix.CEL files were read into R and converted from Log_2_ to linear space. All probes with low expression (<10% of mean) in 10 or more samples were removed. A single probe per gene was selected using collapseRows [23] with default settings and a Pearson correlation (R) between each pair of samples using all 18410 remaining probes was performed. Hierarchical clustering was then performed on the samples using 1-correlation as the distance measure, and the results were plotted in a dendrogram (so a height of 0.02 on the plot means that the samples have R = 0.98).

### qRT-PCR

For qRT-PCR analysis, equal amounts of RNA were assessed using the Brilliant II SYBR green qRT-PCR 1-step master mix (Agilent Technologies). Relative expression values were obtained using the standard curve method and normalized to the *Actb* levels. Amplification efficiencies were calculated using the MxPro software (Stratagene) and were within accepted parameters (80–120%). Data for immunoprecipitates is presented as the fold change versus saline-treated animals. Enrichment is calculated as the IP *versus* input ratio and shows the abundance of the transcript in the targeted cell type (IP) when compared to equal amounts of RNA from the whole testis (input). Sequences of the different primer sets used were obtained from Primerbank (http://pga.mgh.harvard.edu/primerbank/) [24] and can be found in Table S6.

### MA-10 Cell Culture

Cells were cultured as described in [25]. 1×10^5^ cells/ml were seeded onto gelatin-coated 6-well plates and allowed to attach for 2 days. For LH treatment, cells were incubated in serum-free medium containing 1% BSA overnight before the addition of purified human LH (0.2 Units/ml). Cells were lysed in SDS sample buffer (62.5 mM Tris, pH 6.8, 2% SDS, 10% glycerol) for protein analysis, in RLT buffer (Qiagen) for RNA extraction, or as described in [26] for polysome analysis. Sucrose density gradient fractionation and polysome analysis was performed as described in [20].

### Western Blot Analysis

Protein lysates were quantified using the BCA protein assay (Pierce). After protein determination, 20 ug of protein were separated by SDS-PAGE and transferred onto nitrocellulose membranes. After transfer, membranes were blocked with 5% milk in TBST (Tris-buffered saline containing 0.1% Tween-20) and incubated overnight with the following primary antibodies: anti-Aquaporin 2 (1∶1000; Novus), anti-phospho-S6 (1∶2000; Cell Signaling Technologies) and anti-Beta Actin (1∶50,000; Sigma-Aldrich). After incubation with secondary antibodies (1∶10,000; Jackson ImmunoResearch), membranes were washed in TBST and developed using an enhanced chemiluminescence (ECL) detection system (Pierce).

### Immunohistochemistry (IHC)

Mice were perfused transcardially with Bouin fixative or PBS containing 4% paraformaldehyde and testes were removed, post-fixed overnight and cryoprotected with 30% sucrose in PBS. For cryosectioning, testes were frozen for 5 min in dry ice and sectioned using a freezing microtome (Leica). Frozen sections were mounted onto slides and stored at −80°C until ready for use. For IHC, slides were removed from −80°C and allowed to air dry for a few minutes before transferring into wash buffer (PBS with 0.2% Triton X-100). For quenching of the endogenous peroxidase, slides were immersed into 0.3% H_2_O_2_ in methanol for 30 min, and washed twice before blocking with 2.5% normal blocking serum in wash buffer. After blocking, sections were incubated with the primary antibody (biotin-labeled anti-HA antibody at 1∶1000, Covance; or anti-Aquaporin 2 at 1∶200, Novus) overnight at 4°C, and washed twice before the addition of the R.T.U. VECTASTAIN Elite ABC reagent or ImmPRESS reagent (Vector Labs) for 30 min. After incubation, sections were washed twice for 5 min in PBS and developed using the ImmPACT DAB peroxidase substrate (Vector). HA stained sections were also counterstained with Hematoxylin QS (Vector) before mounting with VectaMount AQ (Vector).

## Results

### Activation of RiboTag in Sertoli or Leydig Cells of the Testis

**Figure 1:**
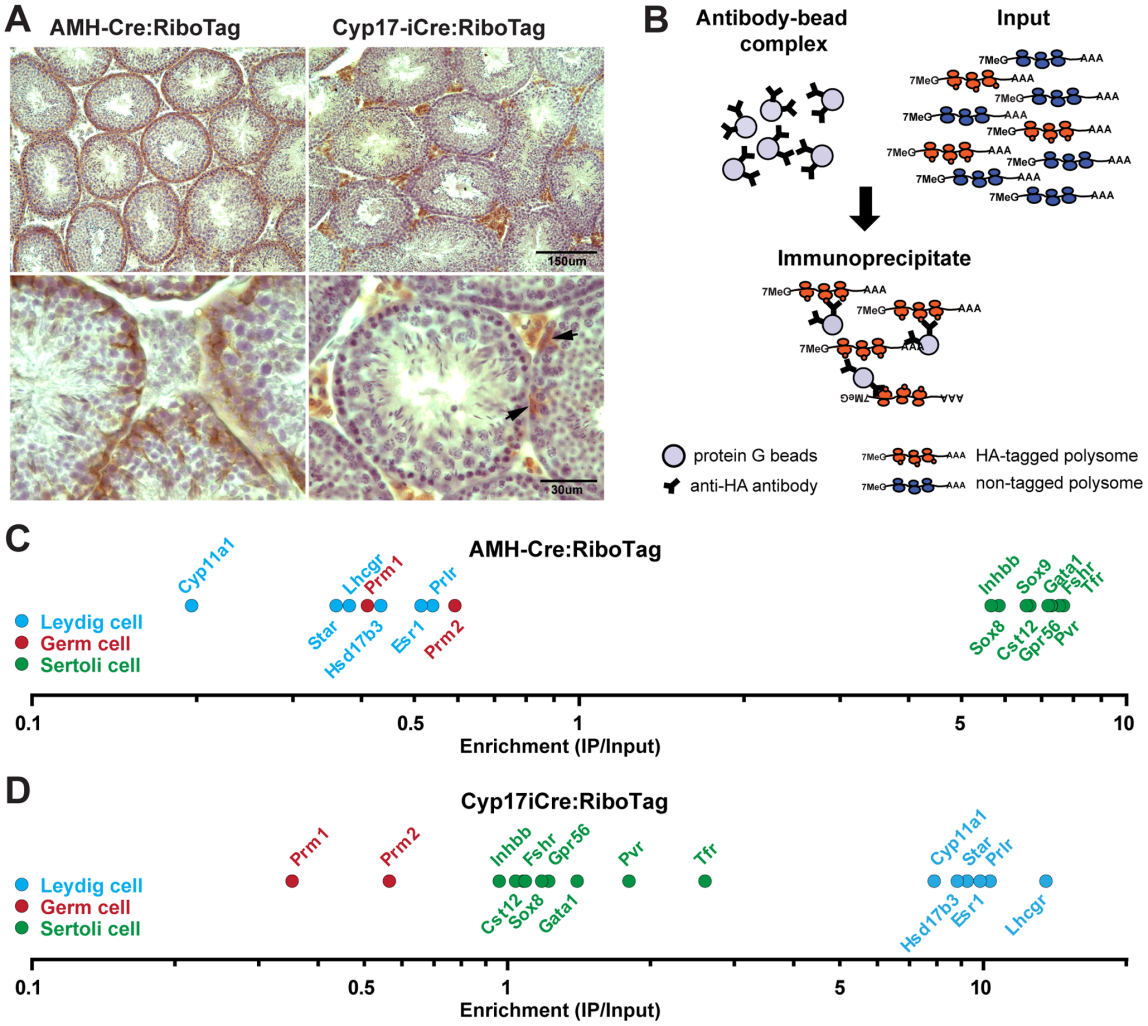
Activation of the RiboTag in Leydig or Sertoli cells of the testis. (A) RiboTag mice were crossed to a Leydig cell-specific Cre line (Cyp17iCre mice) or a Sertoli cell-specific Cre line (AMH-Cre mice) and the RiboTag activation in the cell type of interest was verified by immunohistochemistry using an anti-HA antibody in testis sections. Arrows point to an unexpected RiboTag activation in scattered cells of the tubule in the Cyp17iCre: RiboTag mice. (B) Cartoon depicting the RiboTag assay. Magnetic beads coupled to anti-HA antibodies were added to testis homogenates of Cyp17iCre: RiboTag or AMH-Cre: RiboTag mice (input of the IP) and incubated overnight at 4°C to capture the HA-tagged polysomes from Leydig or Sertoli cells. After incubation, immunoprecipitates were recovered prior to RNA isolation and analysis. (C) Microarray analysis results confirm the enrichment for well-established Sertoli cell-specific transcripts, as well as the negative enrichment for Leydig and germ cell markers in the IPs from AMH-Cre: RiboTag mice. (D) Similar analysis in Cyp17iCre: RiboTag IPs revealed a significant enrichment for Leydig cell-specific transcripts and a negative enrichment for germ cell-specific mRNAs. Sertoli cell specific transcripts did not show a significant negative enrichment consistent with the RiboTag activation in some Sertoli cells. The enrichment was calculated as the ratio of the signal in the IPs to their inputs.

In order to label ribosomes in Sertoli or Leydig cells, AMH-Cre [22] or Cyp17iCre mice [21], respectively, were crossed to RiboTag homozygous mice to obtain double heterozygote Cre: RiboTag offspring. RiboTag activation in the cell type of interest was confirmed by immunohistochemistry for hemmaglutinin (HA) in testis sections of AMH-Cre: RiboTag or Cyp17iCre: RiboTag mice (Fig. 1*A*). HA staining within the seminiferous tubules in AMH-Cre: RiboTag mouse sections was consistent with RiboTag activation in Sertoli cells. In Cyp17iCre: RiboTag mice, robust HA staining was observed in the interstitial spaces of the testis where Leydig cells are located; however, unexpected HA staining in scattered cells of the tubule was also observed (Fig. 1*A*, arrows), suggesting that the RiboTag was also activated in some non-Leydig cell types. Once the RiboTag was activated, cell type-specific transcripts were isolated from the total pool of messenger RNAs (input) by an affinity purification method using an anti-HA antibody coupled to protein G magnetic beads as depicted in Fig. 1*B*.

### The RiboTag Assay Reveals Novel Sertoli and Leydig Cell-specific Transcripts

**Figure 2:**
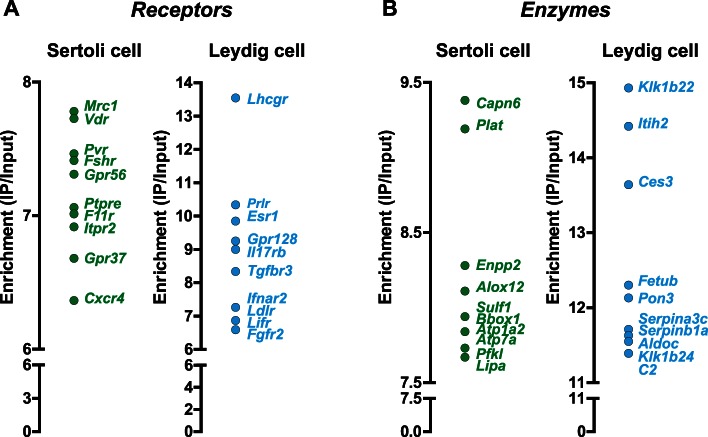
Characterization of cell-specific receptors and enzymes in Sertoli and Leydig cells. Microarray analysis data showing the top ten receptors (A) and enzymes (B) with the highest enrichment in Sertoli and Leydig cells. The enrichment value was calculated as the ratio of the signal in the IPs to their inputs.

To identify novel Sertoli and Leydig cell-specific transcripts we performed microarray analysis using equivalent amounts of total RNA extracted from immunoprecipitates (IPs) and inputs from AMH-Cre: RiboTag or Cyp17iCre:RiboTag mouse testis, respectively. In AMH-Cre: RiboTag mouse testis, the IP fraction was highly enriched in Sertoli cell-specific transcripts such as Transferrin (*Tfr*), follicle-stimulating hormone receptor (*Fshr*) and Poliovirus receptor (*Pvr*); while it was significantly de-enriched in Leydig and germ cell-specific transcripts such as the LH receptor (*Lhcgr*), steroidogenic acute regulatory protein *(Star)*, Protamine 1 and 2 (*Prm1* and *Prm2*) (Fig. 1*C*). Using the enrichment data we generated a list of the top 50 Sertoli cell-specific genes (Table S1; a complete list of all genes with fold enrichment ≥2 is shown in Dataset S1). Among these enriched genes, we could identify novel Sertoli cell-specific transcripts coding for receptors, such as the Mannose receptor *Mrc1*, the Vitamin D receptor (*Vdr*), the inositol triphosphate receptor *Itpr2* or the G-protein coupled receptor *Gpr37* (Fig. 2*A*), or enzymes such as Calpain 6 (*Capn6*), the phophodiesterase/phospholipase *Enpp2* or the arachidonate lipooxygenase *Alox12,* among others (Fig. 2*B*). Gene ontology (GO) analysis of Sertoli cell-specific or highly enriched transcripts (those that showed a 5-fold or higher IP/input ratio) identified several overrepresented GO categories (Table S2), such as regulation of cellular compartment movement or regulation of cell migration, consistent with the active involvement of Sertoli cells in the process of migration and differentiation of spermatogonial stem cells from the basal to the adluminal compartment, and cytoskeletal protein and actin binding, which included several transcripts that code for components of the highly specialized structures found in Sertoli cells called ectoplasmic specializations. Furthermore, GO analysis identified a significant enrichment for transcripts involved in sex determination, GTPase regulatory activity, formate-tetrahydrofolate ligase activity and phosphodiesterase 1 activity, pointing to potential novel functions in Sertoli cells.

Similar analysis in Cyp17iCre: RiboTag testis demonstrated enrichment for well-known Leydig cell specific transcripts such as the LH receptor (*Lhcgr*) and *Star*, among others, while germ cell-specific transcripts showed de-enrichment (IP/Input ratio <1; Fig. 1*D*). Unexpectedly, Sertoli cell transcripts did not show negative enrichment, suggesting that the scattered HA-positive cells observed within the tubule are Sertoli cells, underscoring the high sensitivity of the RiboTag approach (Fig. 1*D, and A*, arrows). However, while most of the Sertoli cell-specific transcripts found showed an IP/input ratio of 1–1.7, Leydig cell-specific transcripts were enriched 7-fold or greater, allowing us to identify cell-specific/highly-enriched Leydig cell transcripts. The IP/input ratio analysis in Cyp17iCre: RiboTag testis identified the top 50 Leydig cell-specific (or highly enriched) transcripts (Table S3, complete table of enriched genes is available in Dataset S2); these enriched transcripts included novel receptors such as the IL17 receptor *Il17br*, the G-protein coupled receptor *Gpr128*, the interferon receptor *Ifnar2*, and the low density lipoprotein receptor (*ldlr*), among others (Fig. 2*A*). We also identified novel Leydig cell-specific enzymes such as the carboxylesterase *Ces3*, Fetuin-beta (*Fetub*), the paraoxonase *Pon3* and the kallikrein-related peptidase *Klk1b22* (Fig. 2*B*). Of note, other transcripts of the kallikrein family of serine proteases or the related family of serine protease inhibitors, the serpins, also showed significant enrichment (Table S4). While several members of the kallikrein family have been reported as Leydig cell-specific [27], [28], [29], the kallikrein family member that showed the highest enrichment in our RiboTag experiments, *Klk1b22*, had not been previously identified in Leydig cells. GO analysis of the Leydig enriched transcripts (7-fold or higher) revealed highly significant molecular function and biological process categories related to steroidogenesis (Table S5), such as lipid, alcohol, cellular ketone and organic acid metabolic processes, oxidoreductase activity, steroid dehydrogenase activity and steroid and coenzyme binding, confirming the highly specialized nature of this cell type in the testis. Two transcripts (*Spnb1* and *Etl4*) showed artifactual enrichment in both Cyp17iCre: RiboTag and AMH-Cre: RiboTag IPs, suggesting non-specific IP of these transcripts perhaps due to anti-HA antibody binding to nascent chains coded by these two mRNAs or some other type of non-specific interaction.

### Regulation of Sertoli Cell Transcripts by Testosterone (T) and FSH

To characterize the transcripts regulated by T or FSH in the polysomes of Sertoli cells, AMH-Cre: RiboTag mice were treated with the GnRH antagonists cetrorelix or acyline every 24 h for 3 days prior to T or FSH administration (Text S1). Both GnRH antagonists suppressed T and greatly reduced FSH levels in serum (Fig. S2 *A* and Fig. S3 *A*). However, microarray analysis of the polysome-associated mRNAs after gonadotropin depletion with these two different GnRH antagonists resulted in only minor changes when compared to the effects of gonadotropin withdrawal in Leydig cells (Fig. S1). Administration of testosterone enanthate or highly purified FSH restored the circulating levels of these two hormones (Fig. S2 *A* and Fig. S3 *A*), but only modest effects on Sertoli-enriched genes were seen in both cases (Fig. S2 *B* and Fig. S3 *B–C*). Few genes were regulated after 4 h of T, and only two, *Slc15a1* and *Kazald1*, were highly enriched in Sertoli cells (Fig. S2 *C* and Fig. S2 *D*). These two transcripts along with two other Sertoli cell enriched transcripts that showed increased polysome association after T stimulation were further confirmed by qRT-PCR analysis (Fig. S2 *D*).

We assayed changes in FSH induced mRNA association with Sertoli cell polysomes at 1 hr to detect rapid effects on translation and induction of immediate early genes. Messenger RNA changes at 4 hr after FSH were assayed to capture more slowly induced changes and to allow comparison with previously published data in hypogonadal mice after 4 hr of FSH stimulation [15]. After 1 h of FSH stimulation, none of the upregulated transcripts were Sertoli cell enriched, suggesting that these transcripts are either not expressed or expressed at very low levels in Sertoli cells (Fig. S3 *B*). Transcripts enriched in Sertoli cells, such as *Eomes*, *Reln* and *Chst2,* among others, showed decreased polysome association after 1 h of FSH (Fig. S3 *B* and *D*). After 4 h of FSH, the transcript response in Sertoli cells was minor and only 9 Sertoli-enriched transcripts showed changes greater than 1.5 fold in the immunoprecipitated polysome fraction (Fig. S3 *C* and *E*).

### Early Leydig Cell mRNA Profile after LH Restoration

**Figure 3:**
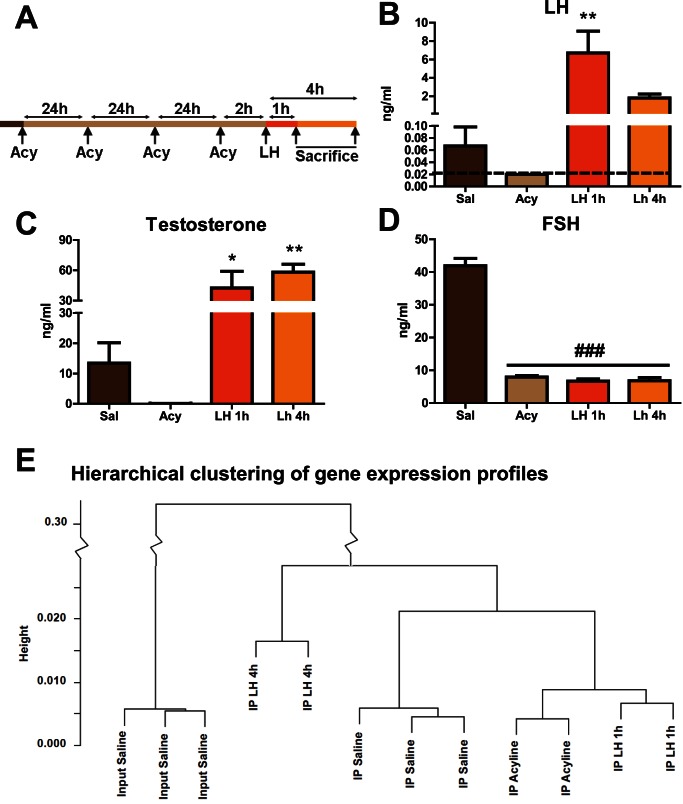
Experimental design, serum hormone levels, and hierarchical clustering of the microarray data after treatments. Experimental design is shown in panel (A). Cyp17iCre: RiboTag mice were injected with the GnRH antagonist acyline (300 ug) for 4 d before a single intraperitoneal injection of LH (2 units). Mice were sacrificed at 1 h and 4 h post-injection and serum levels of LH (B), T (C) and FSH (D) were determined by RIA or EIA (n = 4, from two independent experiments). Graphs show the mean ± SEM. Statistical analysis was performed using One-way Analysis of Variance (ANOVA) with Newman-Keuls multiple comparison post-hoc test. *** p<0.001, ** p<0.01, * p<0.05 *vs* acyline, ### p<0.001 *vs* saline. Dashed line in (B) shows the lower reportable limit. All acyline samples were below this limit. (E) Hierarchical clustering of the microarray data. Samples were clustered using all genes present in at least one sample. The y-axis represents the correlation distance (1-R) between each pair of samples at the nearest branch point between the two samples. Note that all samples from the same treatment group together.

To identify novel transcripts involved in the response of Leydig cells to LH *in vivo*, Cyp17iCre: RiboTag mice were treated as described in Fig. 3*A*. Mice received a subcutaneous injection of the GnRH antagonist acyline every 24 h for 4 days, followed by a single intraperitoneal injection of purified LH (2 units) 2 h after the last acyline injection. To characterize the transcripts involved in the immediate-early, and the intermediate-late response to LH, mice were sacrificed at 1 h and 4 h after LH administration. Repeated injections of acyline completely abolished LH and T serum levels, and reduced FSH levels by 84% (Fig. 3*B*–*D*). As expected, a single injection of LH (2 units) significantly increased LH and T serum levels (Fig. 3*B and C*), but did not increase FSH serum levels (Fig. 3*D*), confirming the lack of significant FSH contamination in the LH preparation.

**Figure 4:**
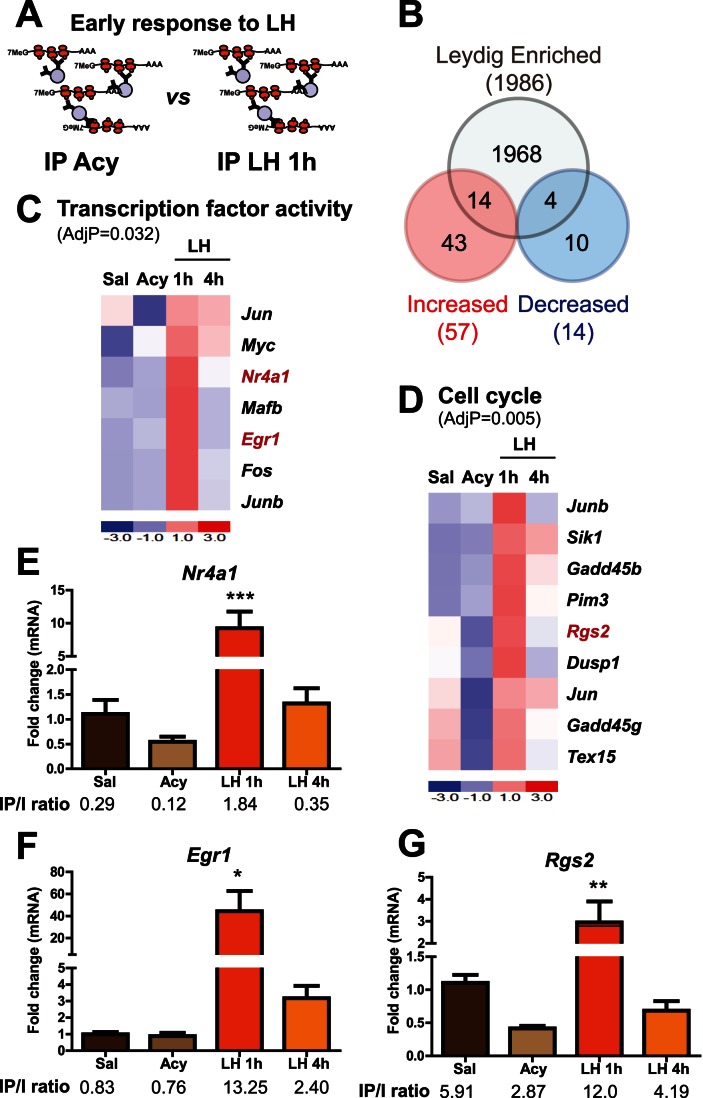
*In vivo* Leydig cell translational profile after 1 h of LH administration. (A) Cartoon shows the two experimental groups compared by microarray analysis. (B) Venn diagrams showing the number of mRNAs regulated after 1 h of LH administration (1.5 fold or higher) when compared to the acyline group in the IPs of Cyp17iCre: RiboTag mice by microarray analysis. Values in the intersection are the number of transcripts regulated by LH and enriched (IP/input >2 in untreated mice) in Leydig cells. GO analysis was performed on the transcripts that showed a fold increase of 1.5 or higher after 1 h of LH stimulation. Transcription factor activity (C) and cell cycle (D) were identified as significant categories. Heat maps show the regulation by microarray analysis of the transcripts included in these GO categories. AdjP: Adjusted p-value. Some transcripts (shown in red) were further confirmed by qRT-PCR because of their novelty or significance (E–F and G). Graphs show qRT-PCR confirmation for *Nr4a1*, *Egr1* (E–F) and *Rgs2* (G) in the IPs of Cyp17iCre: RiboTag mice treated with saline, acyline, acyline+LH for 1 h and acyline+LH for 4 h (n = 4, from two independent experiments). Statistical analysis was performed using One-way Analysis of Variance (ANOVA) with Newman-Keuls multiple comparison post-hoc test. *** p<0.001, ** p<0.01 and * p<0.05 *vs* acyline. IP *versus* input ratio (Enrichment) was assessed by qRT-PCR and shows the abundance of the transcript in Leydig cells. Data are the mean±SEM.

After treatments, the HA-tagged polysomes were recovered and microarray analysis performed. Hierarchical clustering of Affymetrix array data from all treatment groups correctly distinguished individual groups based upon their gene expression profiles, providing additional confidence in the experimental paradigm (Fig. 3*E*). Comparison of IP pellets from acyline treated versus 1 hr LH treated samples revealed 71 specific mRNAs with altered ribosome association at this time point, with most of the transcripts being increased (Fig. 4*A–B*, transcripts with a 2-fold or higher increase after 1 h of LH administration are shown in Fig. S4 *A*). A subsequent enrichment analysis (IP/input ratio in saline-treated animals) identified those transcripts enriched in Leydig cells (Fig. S4B). None of the transcripts positively regulated by LH are Leydig cell-specific (Enrichment >7). GO analysis of the transcripts with a 1.5 or higher fold change identified transcription factor activity and cell cycle as two of the top overrepresented categories (Fig. 4*C and D*). One particular group of transcription factors showed a dramatic increase in ribosome association after 1 h of LH stimulation, returning to basal levels at 4 h of LH stimulation (Fig. 4*C*). Two of the most strongly induced factors, *Nr4a1* and *Egr1*, were validated by qRT-PCR analysis (Fig. 4*E–F*). Enrichment analysis indicated that *Nr4a1* and *Egr1* transcripts were either de-enriched (IP/input <1), or not enriched (IP/input ∼1) in control (saline treated) Leydig cells, respectively, but strongly induced in Leydig cells after 1 h of LH treatment, which is reflected in the increased enrichment ratio in 1 h LH samples (Fig. 4*E–F*). This is the expected result for genes that are expressed in multiple cell types in the testis but are LH regulated in Leydig cells (a more detailed enrichment analysis is illustrated in Fig. S5). Regarding the cell cycle GO category, *Rgs2* was identified as an interesting candidate because of its potential involvement in regulating the LH receptor. IP *versus* input analysis revealed that *Rgs2* was the only transcript of the *Rgs* family significantly enriched in Leydig cells (Fig. S6 *A*). Furthermore, qRT-PCR analysis of the IP fractions after acyline and LH treatment also confirmed that *Rgs2* levels were decreased by acyline, and that LH stimulation for 1 h significantly increased *Rgs2* transcript association with ribosomes. Enrichment ratios (IP/input) in acyline and 1 h LH samples also indicated that the increase in *Rgs2* was specific to Leydig cells, as the enrichment ratio increased in the 1 h LH sample and decreased in the acyline samples (Fig. *4G*).

### Late Leydig Cell mRNA Profile after LH Restoration

**Figure 5:**
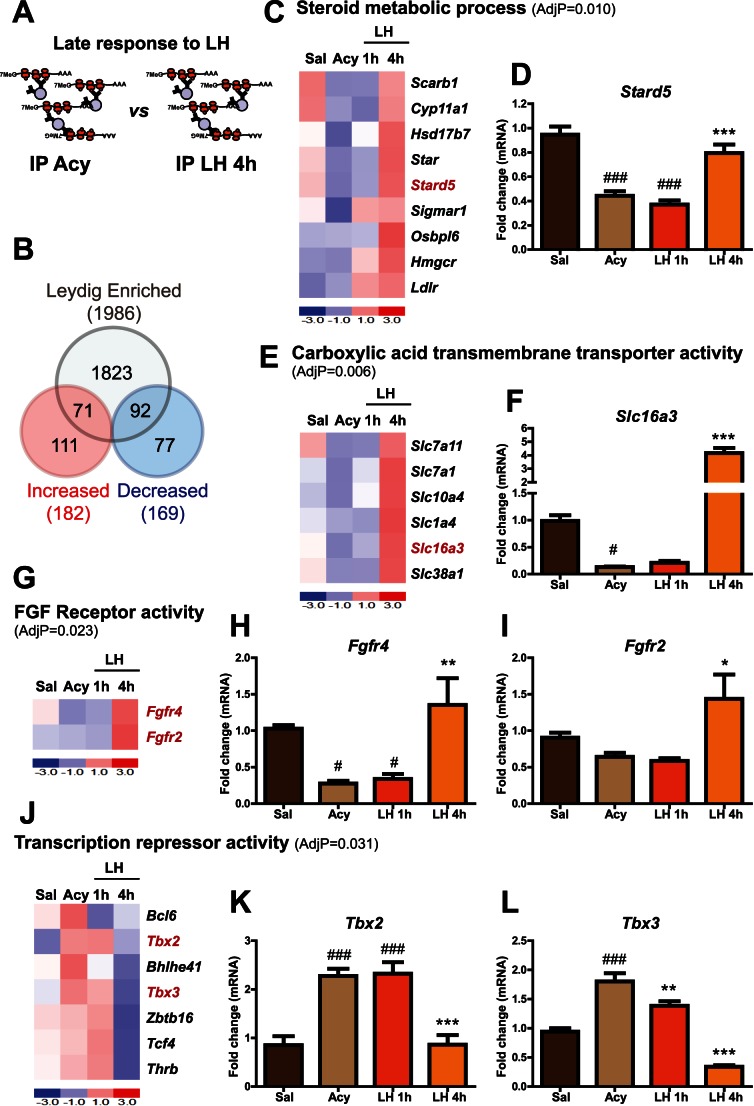
*In vivo* Leydig cell translational profile after 4 h of LH administration. (A) Cartoon showing the two experimental groups compared by the microarray analysis. (B) Venn diagrams showing the number of mRNAs regulated in the IPs of Cyp17iCre: RiboTag mice after 4 h of LH administration (1.5 fold or higher) when compared to the acyline group by microarray analysis. Values in the intersection are the number of transcripts regulated by LH and enriched (IP/input >2 in untreated mice) in Leydig cells. GO analysis was performed on the transcripts that showed a fold increase of 1.5 or higher after 4 h of LH stimulation. Steroid metabolic process (C), carboxylic acid transmembrane transporter activity (E), FGF receptor activity (G) and transcription repressor activity (J) were identified as significant GO categories. Heat maps show the regulation by microarray analysis of the transcripts included in these GO categories. AdjP: Adjusted p-value. Some transcripts (shown in red) were further confirmed by qRT-PCR because of their novelty or significance (D, F, H–I and K–L). Graphs show qRT-PCR confirmation for *Stard5* (D), *Slc16a3* (F), *Fgfr4*, *Fgfr2* (H–I), *Tbx2* and *Tbx3* (K–L) in the IPs of Cyp17iCre: RiboTag mice testis treated with saline, acyline, acyline+LH for 1 h and acyline+LH for 4 h (n = 4, from two independent experiments). Statistical analysis was performed using One-way Analysis of Variance (ANOVA) with Newman-Keuls multiple comparison post-hoc test. ### p<0.001, # p<0.05 *vs* saline; *** p<0.001, ** p<0.01, * p<0.05 *vs* acyline. Enrichment (IP *versus* input ratio) for the analyzed transcripts in saline-treated animals by qRT-PCR analysis was the following: 17.4±1.3 for *Slc16a3,* 21.6±2.3 for *Star*, 9.8±1.3 for *Stard5*, 8.0±0.7 for *Scarb1,* 12.0±1.2 for *Fgfr2*, 15.9±1.2 for *Fgfr4,* 4.1±0.9 for *Tbx2*, and 13.3±1.6 for *Tbx3*. Data are the mean±SEM.

Microarray analysis of transcripts associated with HA-tagged polysomes in Cyp17iCre: RiboTag mice testis after 4 h of LH stimulation compared to those treated with acyline revealed 351 regulated mRNAs (Fig. 5*A–B*, transcripts with a 2 fold or higher increase after LH stimulation are shown in Fig. S7 *A*). Some of them were also identified as highly enriched in Leydig cells (Fig. S7 *B*). Consistent with the known physiological role of Leydig cells, GO analysis of the transcripts with increased ribosome association (1.5 fold or higher) revealed that steroidogenesis-related categories were overrepresented (Fig. 5C). Our results confirmed previously described transcripts, such as *Star, Cyp11a1* and *Scarb1*, but also revealed that the *Star*-related transcript *Stard5* is regulated by LH. Acyline treatment significantly reduced the ribosome-association of *Stard5*, which then increased significantly with 4 h of LH treatment. qRT-PCR analysis confirmed the microarray results (Fig. 5*D*). Enrichment analysis by microarray showed that *Star* (also known as *Stard1*), *Stard4* and *Stard5* were the only transcripts in the *Stard* family that showed a significant enrichment in the IP when compared to their inputs (Fig. S6 *B*). The enrichment for *Stard5* in Leydig cells was further confirmed by qRT-PCR (Fig. 5*D* *legend*).

Importantly, our approach allowed us to identify novel overrepresented categories after 4 h of LH exposure such as carboxylic acid transmembrane transporter activity and FGF receptor activity (Fig. 5*E and G*). Most of the transcripts related to carboxylic acid transmembrane transporter activity were amino acid transporters (*Slc7a1, Slc38a1, Slc7a11, Slc1a4)*, yet the most prominent was the lactic acid and pyruvate proton-linked transporter *Slc16a3* (monocarboxylic acid transporter 4; MCT4). *Slc16a3* was also significantly enriched in the IPs as assessed by microarray and qRT-PCR analysis (Fig. 5*F* *legend and* Fig. S6 *C*). qRT-PCR analysis confirmed that the ribosome-association of this transcript was reduced after acyline treatment and dramatically increased after 4 h of LH treatment (Fig. 5*F*). In addition, two members of the FGF receptor activity family transcripts (*Fgfr4, Fgfr2*) showed increased ribosome-association in the microarray analysis after 4 h of LH treatment (Fig. 5*G*) and were significantly enriched in Leydig cell polysomes (Fig. S6 *D*). qRT-PCR analysis confirmed these results (Fig. 5*H–I*). Finally, transcripts involved in the negative regulation of adenylate cyclase activity were also induced after 4 h of LH, which included the sphingosine-1-phosphate (S1P) receptors *S1pr1* and *S1pr3* (Fig. S7C), suggesting a possible role of S1P in the modulation of LH signaling in Leydig cells.

### Negative Regulation of Leydig Cell Transcripts after LH Administration

LH administration also resulted in decreased ribosome-association of a group of mRNAs which was more evident after 4 h of LH treatment compared with 1 h LH (Fig. 4*B* and 5*B*) and included novel Leydig cell-specific (or highly enriched) transcripts (Fig. S7 *B*). mRNAs that showed a 2-fold or greater decrease after 4 h of LH administration are listed in Fig. S7 *E*. Among them, *Fetub* was confirmed by qRT-PCR to be highly specific to Leydig cells and significantly reduced after LH treatment (Fig. S7 *D*).

Transcriptional repressor activity was among the most significant GO categories of transcripts that were reduced after 4 h of LH (Fig. 5*J*). *Tbx2* and *Tbx3* were two transcripts with transcription repressor activity that responded robustly to acyline and LH. qRT-PCR analysis confirmed that acyline significantly increased the ribosome-association of *Tbx2* and *Tbx3,* which was reversed with 1 h of LH treatment for *Tbx3* and 4 h of LH treatment for *Tbx2* (Fig. 5*K–L*). qRT-PCR results also confirmed that these transcripts were significantly enriched in Leydig cells (Fig. 5*K–L*, *legend*) and similar analysis using the microarray data showed that these transcripts were the only *Tbx* family members that showed a significant enrichment in Leydig cells (Fig. S6 *E*). Another significant GO category of transcripts with decreased polysome-association after 4 h of LH stimulation included those with ligand-dependent nuclear receptor activity, and contained the thyroid hormone receptor *Thrb*, the Rev-Erb receptors *Nr1d1* and *Nr1d2,* and the orphan receptor *Nr0b2* (Fig. S7 *F*). These nuclear receptors are also known to act as transcriptional repressors.

### Regulation of Aquaporin 2 by LH in Leydig Cells

**Figure 6:**
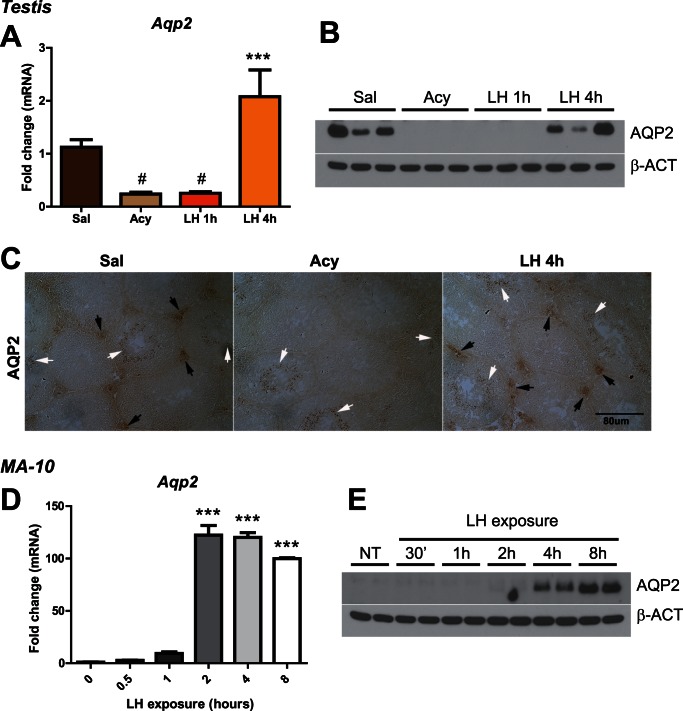
Aquaporin 2 is actively translated in Leydig cells after LH stimulation. (A) qRT-PCR analysis of *Aqp2* mRNA in the IPs of Cyp17iCre: RiboTag mice treated with saline, acyline, acyline+LH for 1 h and acyline+LH for 4 h (n = 4, from two independent experiments). Statistical analysis was performed using One-way Analysis of Variance (ANOVA) with Newman-Keuls multiple comparison post-hoc test. # p<0.05 *vs* saline; *** p<0.001 *vs* acyline. Enrichment (IP *versus* input ratio) for *Aqp2* in saline-treated animals was 17.9±1.7 by qRT-PCR analysis. Data are the mean±SEM. (B) Western blot analysis of aquaporin 2 in testis lysates of mice treated with saline, acyline, acyline+LH for 1 h and acyline+LH for 4 h. Membrane was reprobed with an anti-beta-actin antibody to confirm equal loading of the samples. (C) Immunohistochemistry for aquaporin 2 in testis sections of mice treated with saline, acyline and acyline+LH for 4 h. Black arrows show staining in Leydig cells and white arrows show staining in germ cells. (D) qRT-PCR analysis of *Aqp2* mRNA in MA-10 cells after LH stimulation (0.2 u/ml). Statistical analysis was performed using One-way Analysis of Variance (ANOVA) with Newman-Keuls multiple comparison post-hoc test. *** p<0.001 *vs* 0 h. (E) Western blot analysis of aquaporin 2 in MA-10 cell lysates after LH exposure (0.2 u/ml) for different time points.

Microarray analysis of transcripts associated with the HA-tagged polysomes in Leydig cells revealed that aquaporin 2 (*Aqp2*) was strongly regulated by acyline and LH treatment. Under basal conditions, *Aqp2* showed a high enrichment in Leydig cells (Fig. 6*A* legend); however, the enrichment was not equivalent to the most Leydig-cell specific transcripts, suggesting that *Aqp2* was also expressed in other cell types of the testis. The microarray results were confirmed by qRT-PCR analysis, western blot of total testis protein lysates and immunohistochemistry using an anti-aquaporin 2-specific antibody (Fig. 6*A–*C). Immunohistochemistry also revealed staining in the central region of some seminiferous tubules, consistent with our results suggesting that *Aqp2* mRNA was highly enriched but not specific to Leydig cells. We confirmed *Aqp2* expression and regulation by LH in the MA-10 Leydig cell line. LH treatment induced a 120-fold increase in *Aqp2* mRNA after 2 h of LH, which was sustained after 4 h and 8 h of LH treatment (Fig. 6*D*). This increase in *Aqp2* mRNA was directly correlated with a dramatic increase in aquaporin 2 protein (Fig. 6*E*). Our data supports a direct regulation of this water channel by LH. In addition, microarray analysis revealed that *Aqp2* was the only member of the aquaporin family that showed a significant enrichment in Leydig cells (Fig. S6 *F*). These results suggest that Leydig cells may be regulating water transport by an LH/cAMP dependent signaling pathway similar to that observed in the vasopressin-dependent epithelial cells of the kidney collecting ducts. The biological role of this transport in Leydig cell function is unknown.

### LH Increases the Polysome-association of Ribosomal Protein Transcripts

**Figure 7:**
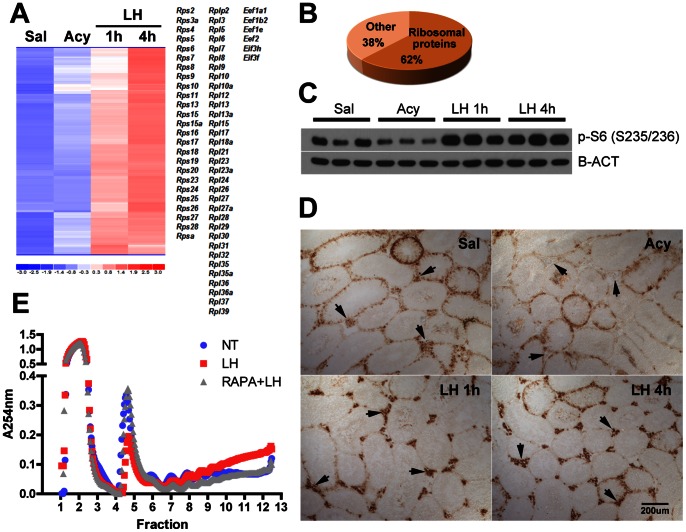
LH induces ribosomal protein translation. (A) Microarray cluster containing the probes that showed increased polysome-association in Cyp17iCre: RiboTag mice after 1 h and 4 h of LH administration. All ribosomal proteins and elongation/initiation factors present in the cluster are listed. Statistical analysis was performed using One-way Analysis of Variance (ANOVA) with Newman-Keuls multiple comparison post-hoc test (p<0.01 *vs* acyline). (B) Pie chart representing the percentage of probes for ribosomal proteins contained in (A). (C) Western blot analysis of phospho-S6 (S235/236) levels in testis lysates after acyline and LH treatment (D) Immunohistochemistry for phospho-S6 ribosomal protein (Ser 235/236) in testis sections of saline, acyline, or acyline+LH-treated mice. Arrows point to the interstitial spaces, where Leydig cells are located. (E) Representative polysome profiles of untreated, LH-treated or rapamycin+LH-treated MA-10 cells. Fractions 1–3 contain the cytosolic fraction, 4–5 the monosome fraction, and fractions 6–12 contain the polysome fraction. Cells were serum-starved overnight and treated for 30 min with Rapamycin (20 nM) or vehicle prior to the addition of LH (0.2 u/ml) for 1 h.

Cluster analysis of the microarray data from the IPs (Text S1) showed an important cohort of transcripts whose polysome-association was increased after 1 h and 4 h of LH administration (Fig. S8 *A*). In addition to several mRNAs coding for elongation and initiation factors, 62% of the probes in this cluster were for ribosomal protein transcripts (Fig. 7*A and B*). One of these mRNAs, *Rps8*, was further confirmed by qRT-PCR (Fig. S8 *B*). This increased presence of ribosomal protein mRNAs in the IPs correlated with an increased phosphorylation of the ribosomal protein S6 at Ser235/236. Western blot analysis of testis lysates showed a decrease in the phosphorylation of the ribosomal protein S6 after acyline treatment, and a dramatic increase after 1 h of LH treatment that was sustained at 4 h (Fig. 7*C*). Immunohistochemical analysis localized these changes to the Leydig cell compartment in the interstitial space (Fig. 7*D*).

We hypothesized that this increased polysome association of mRNAs coding for components of the translational machinery was in response to a stimulatory effect of LH on the translational activity of Leydig cells, and that this effect was mediated by mTOR, as suggested by the increased phosphorylation of S6. To confirm this hypothesis, we assessed the translational status of MA-10 cells after LH stimulation, with or without the addition of the mTOR inhibitor rapamycin (Fig. 7*E*). Polysome profiling analysis of MA-10 cells after LH treatment revealed a decrease in the monosome peak (fractions 4 and 5) with a concomitant increase in the abundance of heavy polysomes (fractions 9–12), consistent with an increased rate of translation initiation. Rapamycin pretreatment prevented the LH effect on the polysome profile confirming the involvement of mTOR signaling in this process. The inhibitory effect of rapamycin on mTOR activity was confirmed by Western blot analysis of phospho-S6 in the lysates (Fig. S8 *C*).

## Discussion

A variety of strategies have been used previously to identify mRNA transcripts specific to somatic cell types in the testis, but these have required purification of Leydig or Sertoli cells using several days of cell culture in conjunction with *in silico* subtraction methods to establish cell-type specific transcripts [30]. Although these approaches have provided valuable insight into testicular biology, they depend upon cell culture techniques that can substantially affect gene expression over several days and are not well adapted to multiple measurements during hormonal regulation. In this paper we have used Cre Driver mice to express tagged ribosomes in either Sertoli or Leydig cells and then directly isolate actively translated mRNAs by polysome immunoprecipitation. Hence, we provide a unique approach to isolate transcripts directly from the complex three-dimensional environment of the testis giving us a snapshot of the translatome in a specific cell type *in vivo*.

RiboTag analysis of AMH-Cre or Cyp17iCre driven cell transcriptomes by enrichment ratio (IP/input) clearly delineated the transcripts specific to Sertoli or Leydig cells, respectively. Enrichment for classical markers was achieved along with identification of previously unidentified cell-specific receptors and enzymes, which will help shed light on the biology of these cell types. In Sertoli cells, the mannose receptor *Mrc1* was one of the novel cell-specific (or highly enriched) receptors described. Although the role of this receptor in Sertoli cells is currently unknown, one of its ligands, the extracellular protease tPA (tissue plasminogen activator, *Plat*) [31], [32] is also Sertoli -cell- specific according to our enrichment analysis. In the testis, the plasminogen activator system is thought to be involved in the degradation of components of the extracellular matrix for the remodeling of the seminiferous tubule during spermatogenesis [33], [34]. Therefore, MRC1 may bind tPA to concentrate its activity in the immediate environment of the Sertoli cell, or participate in the clearance of this protease from the extracellular space [35]. In Leydig cells, enrichment analysis also uncovered new potential targets for the regulation of Leydig cell function by identifying novel cell-specific receptors and enzymes. The scattered Cre-expressing cells in the Cyp17iCre: RiboTag mice that were visualized within the tubule by immunohistochemistry could be identified as Sertoli cells by the mRNA enrichment experiments.

Gene Ontology (GO) analysis of genes enriched in Sertoli and Leydig cells confirmed some well-known functions of these cell types, including regulation of sex determination and the unique actin-containing structures known as ectoplasmic specializations in Sertoli cells [36], [37], [38], and steroid biosynthetic categories in the Leydig cells. However, additional GO categories suggest potential novel biological functions and novel players within these unique cell types. Interestingly, GO analysis in Sertoli cell-enriched genes yielded less statistically robust categories than Leydig-enriched genes, which may be indicative of the more complex cellular role of Sertoli cells in supporting the development of the spermatogonia to become spermatocytes, spermatids and finally mature spermatozoa.

Sertoli cell gene regulation was examined by shutting off endogenous GnRH driven gonadotropin secretion using GnRH receptor antagonists (acyline or cetrorelix) prior to administration of either testosterone (T) or FSH. Analysis of the Sertoli cell ribosome-associated mRNA profile after gonadotropin deprivation demonstrated relatively modest changes and consistent with this, the administration of T or FSH to gonadotropin depleted mice produced changes in only a small number of polysome-associated mRNAs that were Sertoli cell enriched. Among them, we confirmed the regulation of the phosphodiesterase, PDE4D, by FSH as previously described [39], [40]. The modest response of Sertoli cell mRNAs to GnRH antagonists may be due to the residual levels of FSH that remained after antagonist treatment. However, these results suggest that Sertoli cells are not acutely dependent on FSH in contrast to the rapid response of Leydig cells to LH and are consistent with the observation that LH, but not FSH, is essential for fertility in male mice [41], [42].

In contrast to the modest response of Sertoli cell genes to hormonal manipulations, Leydig cell-enriched transcripts responded dramatically to LH. LH stimulation for 1 h induced immediate-early transcription factor activity including *Nr4a1*, *Junb*, *Jun*, *Fos* and *Myc,* in agreement with previous results [43], [44]. In addition we discovered that Egr1 mRNA is strongly induced by LH at this time point. Further analysis of transcripts showing an early response to LH revealed novel candidates for downstream cell signaling such as the regulator of G protein signaling 2 (*Rgs2),* which was induced at 1 h and then returned to baseline at 4 h. G protein-coupled receptors (GPCRs) such as the LH/CG receptor (*Lhcgr*) are negatively regulated by the RGS family and previous reports identified RGS2 as an attenuator of G protein activity during LH receptor signaling in granulosa cells of the ovary [45], [46]. Our results suggest that RGS2 may have similar actions in the Leydig cell. In addition, the fast and transient association of this transcript with polysomes (within 1 h of LH stimulation) is consistent with a role in the regulation of receptor signaling.

Regulation of transcripts involved in steroid metabolism occurred at the later time point (4 h), which suggests that the burst of immediate early gene expression seen at 1 h may be required for subsequent induction of genes involved in steroid biosynthesis at 4 h. Our results confirmed the regulation of several mRNAs involved in the transport and uptake of cholesterol, such as *Star* and *Scarb1*, but, more interestingly, our data revealed that *Star* is not the only START (StAR-related lipid transfer) domain-containing gene to be regulated by LH. *Stard5*, another START-containing transcript, is also enriched in Leydig cells and regulated by LH. It has been proposed that StarD5 may act as a soluble cholesterol transporter shuttling cholesterol from intracellular stores to various membranes such as the plasma membrane or the endoplasmic reticulum (ER) [47]. Thus, in the Leydig cell, StarD5 may be mediating intracellular trafficking of cholesterol. Our results showing that acyline decreases and LH restores *Stard5* transcripts in Leydig cells differs from results using MA-10 cells [48]. Other studies using the Leydig cell tumor line, MA-10, have not always been in agreement with *in vivo* or primary cell culture studies [49], [50] highlighting the importance of using *in vivo* physiological approaches.

The Fibroblast Growth Factor (FGF) family of ligands and its receptors are another category of signaling molecules that play important roles in development of the primitive gonad but also appear to have roles in the fetal, immature and adult testis [51], [52], [53]. Our results demonstrate that in adult Leydig cells, LH signaling is required for the maintenance of *Fgfr2* and *Fgfr4* expression. It has been suggested (using cell culture systems) that FGF signaling in adult and fetal Leydig cells is only responsible for LH-independent steroidogenesis, with no effects on LH/hCG-directed steroid synthesis [54], [55]. Our results suggest that in a physiological environment, FGF signaling via FGFR2 and FGFR4 may be responsive to LH levels.

We have also described the transcripts that undergo negative regulation after LH stimulation. The T-box transcription factors *Tbx2* and *Tbx3* showed increased polysome association when gonadotropin tone was reduced with acyline, and decreased again when LH was restored. *Tbx2* is highly expressed in P0 testis in the interstitium compartment and then decreases below detection at P14 as measured by immunohistochemistry [56]. Leydig cells are a major cell component of the interstitium and our more sensitive mRNA assays demonstrate that both *Tbx2* and *Tbx3* continue to be expressed in adult Leydig cells where they are repressed by the presence of LH. The role of *Tbx2* or *Tbx3* in adult male gonads is unknown since knockout of these genes results in embryonic lethality [57], [58], [59].

Our results have also uncovered specific regulation of the water channel, AQP2, by LH in the Leydig cell. In contrast to a previous study suggesting that *Aqp2* transcripts were restricted to germ cells of the mouse testis [60], our data revealed that *Aqp2* transcripts are in fact highly enriched in Leydig cells and dramatically regulated by LH. This discrepancy may be explained by the indirect method of assessing *Aqp2* expression using a transgenic mouse with a modified Cre recombinase cassette expressed under the control of a 14 kb fragment of the human *Aqp2* promoter [60]. Although the biological significance of AQP2 expression in Leydig cells is unknown, it is well established that Leydig cells have a prominent role in the regulation of the interstitial fluid volume and hCG has been to shown to directly regulate interstitial fluid volume through its actions on these cells [61], [62]. It is also possible that the role of AQP2 in Leydig cells may be cell-autonomous and related to intracellular changes in fluid dynamics in the Leydig cell as these cells become metabolically more active and are synthesizing steroids.

Finally, our results also provide evidence for LH-dependent regulation of the translational apparatus and thus protein synthesis in Leydig cells. The increased ribosome association of a large number of ribosomal protein transcripts as well as several elongation and initiation factor transcripts after LH stimulation suggests a potent stimulatory effect of LH on the translational machinery of the Leydig cell. This increased ribosome association of components of the translational apparatus appears to be mTOR-dependent in agreement with previous reports describing the regulation of protein synthesis by mTOR functioning within the mTORC1 complex [63]. Messenger RNAs coding for ribosomal proteins and elongation/initiation factors are characterized by the presence a 5′ terminal oligopyrimidine tract (referred to as 5′TOP mRNAs) and these transcripts have recently been confirmed to be regulated by mTORC1 at the translational level [64], although there are conflicting results [65]. Our results demonstrate that LH induces the activation of mTOR *in vivo* as assessed by the phosphorylation of the ribosomal protein S6, which correlates with an increased ribosome-association of 5′TOP mRNAs. Although S6 phosphorylation is a reliable marker for mTOR activity, this phosphorylation is not responsible for the translational efficiency of 5′TOP mRNAs [65], [66], as originally thought [67]. Since ribosomal proteins transcripts are regulated at the translational level in higher eukaryotes [68], [69], [70], the RiboTag mouse model is uniquely suited to the identification of this type of regulation**.**

Much of the future work to be carried out in biomedical research will rely on technology that provides cell-type-specific resolution combined with genome-scale analysis of gene expression changes occurring in unique cell types in physiologically meaningful contexts [71], [72], [73]. Toward that end, we have used the RiboTag mouse model combined with microarray analysis to analyze the translatome in two critical somatic cell types of the testis, the Sertoli and Leydig cells, in a paradigm where the hormonal milieu has been fundamentally altered. We have demonstrated the advantages of the technique in allowing rapid enrichment of translated mRNAs specific to each cell type and the application of this technique to detect dynamic changes in the association of particular transcripts with the translational machinery during physiological regulation in the *in vivo* setting.

## Supporting Information

Figure S1 **Sertoli and Leydig cell response to gonadotropin deprivation.** Venn diagrams showing the number of transcripts with increased or decreased polysome association (1.5 fold or higher) after treatment with the GnRH antagonists cetrorelix (A) or acyline (B) in AMH-Cre: RiboTag mice, or with acyline in Cyp17iCre: RiboTag mice (C) by microarray analysis. The number of transcripts regulated by GnRH antagonist administration and enriched (IP/input >2 in untreated mice) in the cell type of interest is shown in the intersection.
(EPS)

Figure S2 ***In vivo*** **Sertoli-cell translational profile after testosterone (T) administration.** (A) T and FSH serum levels in AMH-Cre: RiboTag mice treated with saline, Cetrorelix or Cetrorelix+testosterone (T) (n = 3). Cetrorelix (50 ug) was given s.c. for 3 d before a single i.p. injection of testosterone enanthate (10 mg). Serum was obtained 4 h after the T administration. (B) Venn diagram showing the number of transcripts regulated after T administration (1.5 fold or higher) when compared to the Cetrorelix group by microarray analysis. Values in the intersection are the number of transcripts regulated by T and enriched (IP/input >2 in untreated mice) in Sertoli cells. Table (C) lists these transcripts with their respective fold change and enrichment values. (D) qRT-PCR confirmation of microarray results (n = 3). Enrichment by qRT-PCR was 2.3±0.2 for *Pyroxd2*, 5.8±0.5 for *Kazald1*, 8.0±0.5 for *Slc15a1* and 1.5±0.1 for *Myof*. Data are the mean±SEM. Statistical analysis was performed using One-way Analysis of Variance (ANOVA) with Newman-Keuls multiple comparison post-hoc test. **###** p<0.001, **##** p<0.01 and **#** p<0.05 *vs* saline; *** p<0.001, and * p<0.05 *vs* acyline.
(EPS)

Figure S3 **Sertoli cell translational profile after FSH administration.** (A) Serum levels for FSH and T after acyline and FSH administration (n = 3). Acyline was given for 3 d before FSH (1 u) administration for 1 h and 4 h. Data are the mean±SEM. Statistical analysis was performed using One-way Analysis of Variance (ANOVA) with Newman-Keuls multiple comparison post-hoc test. **##** p<0.01 *vs* saline and *** p<0.001 *vs* acyline. (B–C) Venn diagrams showing the number of transcripts regulated after 1 h or 4 h of FSH administration (1.5 fold or higher) when compared to the acyline group by microarray analysis. Values in the intersection are the number of transcripts regulated by FSH and enriched (IP/input >2 in untreated mice) in Sertoli cells. Tables (D–E) list these transcripts with their respective fold change and enrichment values.
(EPS)

Figure S4 **Leydig cell translational profile after 1 h of LH administration.** (A) Heat map showing the regulation of transcripts with a 2-fold or higher increase after 1 h of LH stimulation (*versus* acyline treatment) in the Cyp17iCre: RiboTag IPs by microarray analysis. Only two transcripts (*Bcl6* and *Dppa4*) showed a two-fold or greater decrease after LH stimulation. Table (B) lists the Leydig-cell enriched mRNAs (2-fold or higher under basal conditions) that showed a 1.5 or higher fold change after 1 h of LH administration.
(EPS)

Figure S5 **qRT-PCR analysis of transcripts expressed at different levels in Leydig cells using inputs and IPs from Cyp17iCre: RiboTag mice.** Mice were treated with saline, acyline, acyline+LH for 1 h and acyline+LH for 4 h (n = 4, from two independent experiments). Notice that analysis of the IP fraction allows for the detection of LH-induced changes in transcripts with low expression in Leydig cells (when compared to other cell types in the testis), and results in higher signal and more statistical significance for the transcripts that are enriched or specific to the targeted cell type. Data are the mean±SEM. Statistical analysis was performed separately for each group (inputs or IPs) using One-way Analysis of Variance (ANOVA) with Newman-Keuls multiple comparison post-hoc test. ### p<0.001, # p<0.05 *vs* saline; *** p<0.001, ** p<0.01, * p<0.05 *vs* acyline.
(EPS)

Figure S6 **Enrichment analysis.** Microarray analysis data of different transcripts from the *Rgs* (A), *Stard* (B), MCT (C), *Fgfr* (D), *Tbx* (E) and *Aqp* (F) family presented as fold change in the IPs *versus* the inputs (Enrichment) in untreated Cyp17iCre: RiboTag mice (n = 3). Data are the mean±SEM.
(EPS)

Figure S7 **Leydig cell translational profile after 4 h of LH administration.** (A) Heat map showing the regulation of transcripts with a 2-fold or higher increase after 4 h of LH administration (*versus* acyline treatment) in the Cyp17iCre: RiboTag IPs by microarray analysis. (B) Table shows the Leydig cell-specific (or highly enriched) transcripts (6-fold or higher under basal conditions) that showed a 1.5 or higher fold change after 4 h of LH stimulation. (C) Heat map showing the regulation of the sphingosine-1-phosphate receptors *S1pr1* and *S1pr3* by microarray analysis in Cyp17iCre: RiboTag mice IPs after treatment with saline, acyline, acyline+LH for 1 h and acyline+LH for 4 h. (D) qRT-PCR confirmation of microarray results for *Fetub* in IPs from Cyp17iCre: RiboTag mice treated with saline, acyline, acyline+LH for 1 h and acyline+LH for 4 h (n = 4, from two independent experiments). Statistical analysis was performed using One-way Analysis of Variance (ANOVA) with Newman-Keuls multiple comparison post-hoc test. ** p<0.01 *vs* acyline. Enrichment (IP *versus* input ratio) by qRT-PCR analysis for *Fetub* in saline-treated animals was in the Leydig cell-specific range (20.8±2.7). (E) Heat map of transcripts that show a 2-fold or greater decrease after 4 h of LH (*versus* acyline treatment) in the Cyp17iCre: RiboTag IPs by microarray analysis. (F) Heat map showing the regulation of transcripts involved in ligand-dependent nuclear receptor activity.
(EPS)

Figure S8 **Cluster analysis, Rps8 confirmation and phospho-S6 levels.** (A) Cluster analysis of the microarray data obtained from IPs of Cyp17iCre: RiboTag mice treated as described. Transcripts that were significantly different between groups (p<0.01 using One-way Analysis of Variance (ANOVA)) were grouped into different clusters according to their response to the treatments. The cluster that contained a significant number of probes for ribosomal proteins (and elongation and initiation factors) is highlighted. (B) qRT-PCR confirmation of *Rps8* levels in IPs from Cyp17iCre: RiboTag mice after acyline and LH administration. Data are the mean±SEM. Statistical analysis was performed using One-way Analysis of Variance (ANOVA) with Newman-Keuls multiple comparison post-hoc test. * p<0.05 *vs* acyline. (C) Western blot analysis of phospho-S6 ribosomal protein in MA-10 Leydig cells treated with LH (0.2 u/ml) for 1 h, with or without rapamycin (20 nM) pretreatment for 30 min. Cells were serum-starved overnight before treatments.
(EPS)

Table S1 **Top 50 Sertoli cell-specific transcripts.** To determine the top Sertoli cell-specific transcripts, microarray analysis of IPs and their respective inputs from AMH-Cre: RiboTag mouse testis (n = 5) was performed and the ratio of the signal in the IP to the input was calculated and expressed as enrichment.
(DOCX)

Table S2 **Gene ontology analysis of Sertoli cell-specific or highly enriched transcripts.** Transcripts that showed an enrichment (IP/I) ratio of 5 fold or higher in IPs from AMH-Cre: RiboTag mice testes were analyzed. GO categories with an AdjP value <0.05 are shown.
(DOCX)

Table S3 **Top 50 Leydig cell-specific transcripts.** Leydig cell-specific transcripts were determined as described previously for Sertoli cells. Microarray analysis of Cyp17iCre: RiboTag mouse testis IPs and their respective inputs (n = 3) was performed and the enrichment was calculated as the ratio of the signal in the IPs compared to their inputs.
(DOCX)

Table S4 **Kallikrein and Serpin family members enriched in Leydig cells.** Table shows the members of the Kallikrein or Serpin family that have an enrichment (IP to input ratio) >4 in the Cyp17iCre: RiboTag pellets compared to their inputs by microarray analysis. Listed references confirm Leydig-cell specificity.
(DOCX)

Table S5 **Gene ontology analysis of Leydig cell-specific or highly enriched transcripts**. Transcripts that showed an enrichment (IP/I) ratio of 7 fold or higher in IPs from Cyp17iCre: RiboTag mouse testes were analyzed. GO categories with an AdjP value <0.01 are listed.
(DOCX)

Table S6 **Sequences of primers used for qRT-PCR analysis.**
(DOCX)

Text S1 **Supplemental experimental procedures.**
(DOCX)

Dataset S1 **Sertoli cell enriched genes.** Transcripts that show a fold change >2 when comparing the signal in the IP to their respective inputs (enrichment) by microarray analysis in saline-treated AMH-Cre: RiboTag mice (n = 5) are listed. Statistical analysis was performed using One-way Analysis of Variance (ANOVA, p<0.05).
(XLSX)

Dataset S2 **Leydig cell enriched genes.** Transcripts that show a fold change >2 when comparing the signal in the IP to their respective inputs (enrichment) by microarray analysis in Saline-treated Cyp17iCre: RiboTag mice (n = 3) are listed. Statistical analysis was performed using One-way Analysis of Variance (ANOVA, p<0.05).
(XLSX)
