# Supplementary material for: RiboTag Analysis of Actively Translated mRNAs in Sertoli and Leydig Cells In Vivo
Source: PLoS One. 2013 Jun 11;8(6):e66179. doi: 10.1371/journal.pone.0066179 (PMC3679032; doi:10.1371/journal.pone.0066179)
Supplement: Table S1 — Top 50 Sertoli cell-specific transcripts. To determine the top Sertoli cell-specific transcripts, microarray analysis of IPs and their respective inputs from AMH-Cre: RiboTag mouse testis (n = 5) was performed and the ratio of the signal in the IP to the input was calculated and expressed as enrichment. (DOCX) [file pone.0066179.s009.docx]

**Top 50 Sertoli cell-specific transcripts**

| *Symbol* | *Description* | *Enrichment* |
| --- | --- | --- |
| Capn6 | calpain 6 | 9.38 |
| Plat | plasminogen activator, tissue | 9.19 |
| Oit3 | oncoprotein induced transcript 3 | 9.19 |
| Gm648 | predicted gene 648 | 9.19 |
| Itga6 | integrin alpha 6 | 9.00 |
| 4833424O15Rik | RIKEN cDNA 4833424O15 gene | 8.82 |
| Pdzd2 | PDZ domain containing 2 | 8.69 |
| Nid1 | nidogen 1 | 8.57 |
| Hmgn5 | high-mobility group nucleosome binding domain 5 | 8.51 |
| Thbs1 | thrombospondin 1 | 8.34 |
| Mcf2 | mcf.2 transforming sequence | 8.34 |
| Enpp2 | ectonucleotide pyrophosphatase/phosphodiesterase 2 | 8.28 |
| Alox12 | arachidonate 12-lipoxygenase | 8.11 |
| Slc27a6 | solute carrier family 27 (fatty acid transporter), member 6 | 8.11 |
| Etv5 | ets variant gene 5 | 8.06 |
| Sulf1 | sulfatase 1 | 7.94 |
| Frzb | frizzled-related protein | 7.89 |
| Gpc4 | glypican 4 | 7.89 |
| 4933402E13Rik | RIKEN cDNA 4933402E13 gene | 7.89 |
| Sept9 | septin 9 | 7.84 |
| Bbox1 | butyrobetaine (gamma), 2-oxoglutarate dioxygenase | 7.84 |
| Atp1a2 | ATPase, Na+/K+ transporting, alpha 2 polypeptide | 7.84 |
| Mrc1 | mannose receptor, C type 1 | 7.78 |
| Sh2d4a | SH2 domain containing 4A | 7.78 |
| Diap2 | diaphanous homolog 2 (Drosophila) | 7.78 |
| Atp7a | ATPase, Cu++ transporting, alpha polypeptide | 7.73 |
| Vdr | vitamin D receptor | 7.73 |
| Pfkl | phosphofructokinase, liver, B-type | 7.67 |
| Butr1 | butyrophilin related 1 | 7.67 |
| Lipa | lysosomal acid lipase A | 7.67 |
| Slc15a1 | solute carrier family 15 (oligopeptide transporter), member 1 | 7.67 |
| Trf | transferrin | 7.62 |
| Sema6c | sema domain, transmembrane domain (TM), and cytoplasmic domain, (semaphorin) 6C | 7.57 |
| Plekhg1 | pleckstrin homology domain containing, family G member 1 | 7.57 |
| Hpse | heparanase | 7.52 |
| Kif21a | kinesin family member 21A | 7.52 |
| Qpct | glutaminyl-peptide cyclotransferase (glutaminyl cyclase) | 7.52 |
| Sema3c | sema domain, immunoglobulin domain (Ig), short basic domain, secreted, (semaphorin) 3C | 7.46 |
| Acsl4 | acyl-CoA synthetase long-chain family member 4 | 7.46 |
| Mtap1b | microtubule-associated protein 1B | 7.46 |
| Pvr | poliovirus receptor | 7.46 |
| Fuca2 | fucosidase, alpha-L- 2, plasma | 7.46 |
| Daam2 | dishevelled associated activator of morphogenesis 2 | 7.46 |
| Emb | embigin | 7.41 |
| Slc2a12 | solute carrier family 2 (facilitated glucose transporter), member 12 | 7.41 |
| Fshr | follicle stimulating hormone receptor | 7.41 |
| Slc27a3 | solute carrier family 27 (fatty acid transporter), member 3 | 7.41 |
| Frmpd1 | FERM and PDZ domain containing 1 | 7.41 |
| Rhpn2 | rhophilin, Rho GTPase binding protein 2 | 7.36 |
| Cytip | cytohesin 1 interacting protein | 7.36 |
