# Supplementary material for: RiboTag Analysis of Actively Translated mRNAs in Sertoli and Leydig Cells In Vivo
Source: PLoS One. 2013 Jun 11;8(6):e66179. doi: 10.1371/journal.pone.0066179 (PMC3679032; doi:10.1371/journal.pone.0066179)
Supplement: Table S2 — Gene ontology analysis of Sertoli cell-specific or highly enriched transcripts. Transcripts that showed an enrichment (IP/I) ratio of 5 fold or higher in IPs from AMH-Cre: RiboTag mice testes were analyzed. GO categories with an AdjP value <0.05 are shown. (DOCX) [file pone.0066179.s010.docx]

**Gene ontology analysis of Sertoli cell-specific or highly enriched transcripts**

| *Biological process* | *Genes* | *AdjP* |
| --- | --- | --- |
| **Localitzation** | 87 | 4.98E-02 |
| *Regulation of cellular component movement* | 9 | 3.49E-02 |
| *Cell migration* | 17 | 4.98E-02 |
| Regulation of cell migration | 9 | 2.26E-02 |
| **Sex determination** | 5 | 2.26E-02 |
| *Male sex determination* | 4 | 2.91E-02 |

| *Molecular function* | *Genes* | *AdjP* |
| --- | --- | --- |
| **Binding** | 268 | 2.45E-02 |
| *Nucleotide binding* | 68 | 2.67E-02 |
| Purine ribonucleotide binding | 61 | 1.70E-02 |
| *Protein binding* | 161 | 5.20E-03 |
| Cytoskeletal protein binding | 21 | 8.90E-03 |
| Actin binding | 18 | 5.20E-03 |
| **Catalytic activity** | 141 | 1.70E-02 |
| *Formate-tetrahydrofolate ligase activity* | 2 | 1.70E-02 |
| *Phosphodiesterase I activity* | 2 | 1.70E-02 |
| *Active transmembrane transporter activity* | 18 | 8.90E-03 |
| sodium potassium-exchanging ATPase activity | 4 | 5.20E-03 |
| *Enzyme regulator activity* | 30 | 8.90E-03 |
| Nucleoside-triphosphatase regulator activity | 18 | 2.67E-02 |
| GTPase regulator activity | 18 | 2.67E-02 |
