# Supplementary material for: RiboTag Analysis of Actively Translated mRNAs in Sertoli and Leydig Cells In Vivo
Source: PLoS One. 2013 Jun 11;8(6):e66179. doi: 10.1371/journal.pone.0066179 (PMC3679032; doi:10.1371/journal.pone.0066179)
Supplement: Table S3 — Top 50 Leydig cell-specific transcripts. Leydig cell-specific transcripts were determined as described previously for Sertoli cells. Microarray analysis of Cyp17iCre: RiboTag mouse testis IPs and their respective inputs (n = 3) was performed and the enrichment was calculated as the ratio of the signal in the IPs compared to their inputs. (DOCX) [file pone.0066179.s011.docx]

**Top 50 Leydig cell-specific transcripts**

| *Symbol* | *Description* | *Enrichment* | |
| --- | --- | --- | --- |
| Klk1b22 | kallikrein 1-related peptidase b22 | 14.93 |  |
| Itih2 | inter-alpha trypsin inhibitor, heavy chain 2 | 14.42 |  |
| Ces3 | carboxylesterase 3 | 13.64 |  |
| Lhcgr | luteinizing hormone/choriogonadotropin receptor | 13.55 |  |
| Fetub | fetuin beta | 12.30 |  |
| Pon3 | paraoxonase 3 | 12.30 |  |
| Serpina3c | serine (or cysteine) peptidase inhibitor, clade A, member 3C | 12.13 |  |
| Lrg1 | leucine-rich alpha-2-glycoprotein 1 | 11.71 |  |
| Serpinb1a | serine (or cysteine) peptidase inhibitor, clade B, member 1a | 11.71 |  |
| Aldoc | aldolase C, fructose-bisphosphate | 11.63 |  |
| Klk1b24 | kallikrein 1-related peptidase b24 | 11.55 |  |
| Apoc1 | apolipoprotein C-I | 11.39 |  |
| BC018465 | cDNA sequence BC018465 | 11.39 |  |
| C2 | complement component 2 (within H-2S) | 11.39 |  |
| Spon1 | spondin 1, (f-spondin) extracellular matrix protein | 11.24 |  |
| Pm20d1 | peptidase M20 domain containing 1 | 10.93 |  |
| Ctsh | cathepsin H | 10.85 |  |
| Aldh1a7 | aldehyde dehydrogenase family 1, subfamily A7 | 10.78 |  |
| Aldh1l1 | aldehyde dehydrogenase 1 family, member L1 | 10.70 |  |
| Tcn2 | transcobalamin 2 | 10.70 |  |
| Adhfe1 | alcohol dehydrogenase, iron containing, 1 | 10.63 |  |
| Rhbg | Rhesus blood group-associated B glycoprotein | 10.63 |  |
| Aff2 | AF4/FMR2 family, member 2 | 10.56 |  |
| Cyp2j6 | cytochrome P450, family 2, subfamily j, polypeptide 6 | 10.48 |  |
| Nqo1 | NAD(P)H dehydrogenase, quinone 1 | 10.48 |  |
| Scd1 | stearoyl-Coenzyme A desaturase 1 | 10.48 |  |
| Acsf2 | acyl-CoA synthetase family member 2 | 10.34 |  |
| Adh1 | alcohol dehydrogenase 1 (class I) | 10.34 |  |
| Amy1 | amylase 1, salivary | 10.34 |  |
| Prlr | prolactin receptor | 10.34 |  |
| AU021092 | expressed sequence AU021092 | 10.27 |  |
| Gpt2 | glutamic pyruvate transaminase (alanine aminotransferase) 2 | 10.27 |  |
| Figf | c-fos induced growth factor | 10.13 |  |
| Cd36 | CD36 antigen | 10.06 |  |
| Glb1l2 | galactosidase, beta 1-like 2 | 10.06 |  |
| Lipg | lipase, endothelial | 9.99 |  |
| Slc38a1 | solute carrier family 38, member 1 | 9.99 |  |
| Tspan8 | tetraspanin 8 | 9.99 |  |
| 9230104L09Rik | RIKEN cDNA 9230104L09 gene | 9.92 |  |
| Aldh3a1 | aldehyde dehydrogenase family 3, subfamily A1 | 9.92 |  |
| Esr1 | estrogen receptor 1 (alpha) | 9.85 |  |
| Sardh | sarcosine dehydrogenase | 9.85 |  |
| Phyh | phytanoyl-CoA hydroxylase | 9.78 |  |
| Tkt | transketolase | 9.71 |  |
| Gstk1 | glutathione S-transferase kappa 1 | 9.65 |  |
| Acsm3 | acyl-CoA synthetase medium-chain family member 3 | 9.45 |  |
| Cat | catalase | 9.32 |  |
| Copz2 | coatomer protein complex, subunit zeta 2 | 9.25 |  |
| Gpr128 | G protein-coupled receptor 128 | 9.25 |  |
| Star | steroidogenic acute regulatory protein | 9.25 |  |
