# Supplementary material for: RiboTag Analysis of Actively Translated mRNAs in Sertoli and Leydig Cells In Vivo
Source: PLoS One. 2013 Jun 11;8(6):e66179. doi: 10.1371/journal.pone.0066179 (PMC3679032; doi:10.1371/journal.pone.0066179)
Supplement: Table S4 — Kallikrein and Serpin family members enriched in Leydig cells. Table shows the members of the Kallikrein or Serpin family that have an enrichment (IP to input ratio) >4 in the Cyp17iCre: RiboTag pellets compared to their inputs by microarray analysis. Listed references confirm Leydig-cell specificity. (DOCX) [file pone.0066179.s012.docx]

**Kallikrein and Serpin family members enriched in Leydig cells**

| *Symbol* | *Description* | *Enrichment* | *References* |
| --- | --- | --- | --- |
| Klk1b22 | kallikrein 1-related peptidase b22 | 14.93 |  |
| Klk1b24 | kallikrein 1-related peptidase b24 | 11.55 | ([1](#_ENREF_1)) |
| Klk1b21 | kallikrein 1-related peptidase b21 | 9.00 | ([2](#_ENREF_2)) |
| Klk1b27 | kallikrein 1-related peptidase b27 | 8.34 | ([3](#_ENREF_3)) |
| Klk1b16 | kallikrein 1-related peptidase b16 | 7.78 |  |
| Klk1 | kallikrein 1 | 4.72 |  |
| Serpina3c | serine (or cysteine) peptidase inhibitor, clade A, member 3C (kallikrein binding protein) | 12.13 |  |
| Serpinb1a | serine (or cysteine) peptidase inhibitor, clade B, member 1a | 11.71 |  |
| Serpina3n | serine (or cysteine) peptidase inhibitor, clade A, member 3N | 7.89 |  |
| Serpina3f | serine (or cysteine) peptidase inhibitor, clade A, member 3F | 6.45 |  |
| Serpinb9 | serine (or cysteine) peptidase inhibitor, clade B, member 9 | 5.94 |  |
| Serpina5 | serine (or cysteine) peptidase inhibitor, clade A, member 5 | 4.99 | ([4](#_ENREF_4)) |
| Serping1 | serine (or cysteine) peptidase inhibitor, clade G, member 1 | 4.14 |  |
| Serpinf1 | serine (or cysteine) peptidase inhibitor, clade F, member 1 | 4.06 |  |
| Serpina3h | serine (or cysteine) peptidase inhibitor, clade A, member 3H | 4.03 |  |

1. Matsui H, Takano N, Takahashi T (2005) Characterization of mouse glandular kallikrein 24 expressed in testicular Leydig cells. *Int J Biochem Cell Biol* 37(11):2333-2343.

2. Matsui H, Takahashi T (2001) Mouse testicular Leydig cells express Klk21, a tissue kallikrein that cleaves fibronectin and IGF-binding protein-3. *Endocrinology* 142(11):4918-4929.

3. Matsui H, Moriyama A, Takahashi T (2000) Cloning and characterization of mouse klk27, a novel tissue kallikrein expressed in testicular Leydig cells and exhibiting chymotrypsin-like specificity. *Eur J Biochem* 267(23):6858-6865.

4. Odet F, Verot A, Le Magueresse-Battistoni B (2006) The mouse testis is the source of various serine proteases and serine proteinase inhibitors (SERPINs): Serine proteases and SERPINs identified in Leydig cells are under gonadotropin regulation. *Endocrinology* 147(9):4374-4383.
