# Supplementary material for: RiboTag Analysis of Actively Translated mRNAs in Sertoli and Leydig Cells In Vivo
Source: PLoS One. 2013 Jun 11;8(6):e66179. doi: 10.1371/journal.pone.0066179 (PMC3679032; doi:10.1371/journal.pone.0066179)
Supplement: Table S5 — Gene ontology analysis of Leydig cell-specific or highly enriched transcripts. Transcripts that showed an enrichment (IP/I) ratio of 7 fold or higher in IPs from Cyp17iCre: RiboTag mouse testes were analyzed. GO categories with an AdjP value <0.01 are listed. (DOCX) [file pone.0066179.s013.docx]

| \| **Gene ontology analysis of Leydig cell-specific or highly enriched transcripts**   \| *Molecular function* \| *Genes* \| *AdjP* \| \| --- \| --- \| --- \| \| **Electron carrier activity** \| 8 \| 6.02E-05 \| \| **Catalitic activity** \| 81 \| 2.92E-13 \| \| **Oxidoreductase activity** \| 36 \| 6.32E-18 \| \| *Oxidoreductase activity, acting on the CH-CH group of donnors* \| 6 \| 9.79E-05 \| \| *Oxidoreductase activity, acting on the aldehyde or oxo group of donnors* \| 4 \| 2.40E-03 \| \| *Oxidoreductase activity, acting on the CH-OH group of donnors* \| 9 \| 1.60E-05 \| \| Oxidoreductase activity, acting on the CH-OH group of donnors, NAD or NADPH as acceptor \| 7 \| 4.00E-04 \| \| *Steroid dehydrogenase activity* \| 4 \| 8.00E-04 \| \| Steroid dehydrogenase activity, acting on the CH-OH group od donnors, NAD or NADPH as acceptor \| 4 \| 4.00E-04 \| \| **Binding** \|  \|  \| \| **Steroid binding** \| 4 \| 5.10E-03 \| \| **Cofactor binding** \| 9 \| 5.00E-04 \| \| *Coenzyme binding* \| 8 \| 4.00E-04 \|  \| *Biological process* \| *Genes* \| *AdjP* \| \| --- \| --- \| --- \| \| **Regulation of hormone levels** \| 8 \| 8.00E-04 \| \| **Hormone metabolic process** \| 7 \| 3.00E-04 \| \| *Cellular hormone metabolic process* \| 7 \| 2.93E-05 \| \| **Metabolic process** \| 86 \| 1.20E-05 \| \| **Oxidation/Reduction** \| 34 \| 1.17E-15 \| \| **Lipid metabolic process** \| 30 \| 1.16E-11 \| \| *Cellular lipid metabolic process* \| 29 \| 2.05E-12 \| \| Isoprenoid metabolic process \| 4 \| 5.40E-03 \| \| Fatty acid metabolic process \| 11 \| 2.85E-05 \| \| Steroid metabolic process \| 14 \| 1.01E-08 \| \| Steroid biosynthetic process \| 9 \| 8.82E-07 \| \| Sterol metabolic process \| 7 \| 2.00E-04 \| \| Cholesterol metabolic process \| 7 \| 8.41E-05 \| \| **Alcohol metabolic process** \| 11 \| 1.90E-03 \| \| **Cellular ketone metabolic process** \| 19 \| 9.30E-07 \| \| **Organic acid metabolic process** \| 19 \| 8.26E-07 \| \| *Oxoacid metabolic process* \| 19 \| 8.26E-07 \| \| Carboxylic acid metabolic process \| 19 \| 8.26E-07 \| \| Monocarboxylic acid metabolic process \| 15 \| 8.26E-07 \| \|  \|  \| \| --- \| --- \| --- \| --- \| --- \| --- \| --- \| --- \| --- \| --- \| --- \| --- \| --- \| --- \| --- \| --- \| --- \| --- \| --- \| --- \| --- \| --- \| --- \| --- \| --- \| --- \| --- \| --- \| --- \| --- \| --- \| --- \| --- \| --- \| --- \| --- \| --- \| --- \| --- \| --- \| --- \| --- \| --- \| --- \| --- \| --- \| --- \| --- \| --- \| --- \| --- \| --- \| --- \| --- \| --- \| --- \| --- \| --- \| --- \| --- \| --- \| --- \| --- \| --- \| --- \| --- \| --- \| --- \| --- \| --- \| --- \| --- \| --- \| --- \| --- \| --- \| --- \| --- \| --- \| --- \| --- \| --- \| --- \| --- \| --- \| --- \| --- \| --- \| --- \| --- \| --- \| --- \| --- \| --- \| --- \| --- \| --- \| --- \| --- \| --- \| --- \| --- \| --- \| --- \| --- \| \|  \|  \|  \| \|  \|  \|  \| \|  \|  \|  \| \|  \|  \|  \| \|  \|  \|  \| \|  \|  \|  \| \|  \|  \|  \| \|  \|  \|  \| \|  \|  \|  \| \|  \|  \|  \| \|  \|  \|  \| |  |  |
| --- | --- | --- | --- | --- | --- | --- | --- | --- | --- | --- | --- | --- | --- | --- | --- | --- | --- | --- | --- | --- | --- | --- | --- | --- | --- | --- | --- | --- | --- | --- | --- | --- | --- | --- | --- | --- | --- | --- | --- | --- | --- | --- | --- | --- | --- | --- | --- | --- | --- | --- | --- | --- | --- | --- | --- | --- | --- | --- | --- | --- | --- | --- | --- | --- | --- | --- | --- | --- | --- | --- | --- | --- | --- | --- | --- | --- | --- | --- | --- | --- | --- | --- | --- | --- | --- | --- | --- | --- | --- | --- | --- | --- | --- | --- | --- | --- | --- | --- | --- | --- | --- | --- | --- | --- | --- | --- | --- | --- | --- | --- | --- | --- | --- | --- | --- | --- | --- | --- | --- | --- | --- | --- | --- | --- | --- | --- | --- | --- | --- | --- | --- | --- | --- | --- | --- | --- | --- | --- | --- | --- |
|  |  |  |
|  |  |  |
