# Supplementary material for: RiboTag Analysis of Actively Translated mRNAs in Sertoli and Leydig Cells In Vivo
Source: PLoS One. 2013 Jun 11;8(6):e66179. doi: 10.1371/journal.pone.0066179 (PMC3679032; doi:10.1371/journal.pone.0066179)
Supplement: Table S6 — Sequences of primers used for qRT-PCR analysis. (DOCX) [file pone.0066179.s014.docx]

**Sequences of primers used for qRT-PCR analysis**

|  | *Sequence* | *Amplicon (bp)* |
| --- | --- | --- |
| *Aqp2* FOR | TGGTGGGTTGCCATGTCTC | 113 |
| *Aqp2* REV | GCGGATTTCTACAGGGGTAATCT |  |
| *Capn6* FOR | GGAAGCGTCCACAGGACATTT | 99 |
| *Capn6* REV | TCATTGCCTTGTTCCCCAATC |  |
| *Fetub* FOR | ACTGCCCTAGCCCCATTGA | 132 |
| *Fetub* REV | ACTGGTTCATAGCCTTGGTGA |  |
| *Fgfr2* FOR | AATCTCCCAACCAGAAGCGTA | 142 |
| *Fgfr2* REV | CTCCCCAATAAGCACTGTCCT |  |
| *Fgfr4* FOR | CCTTCCACGGGGAGAATCG | 113 |
| *Fgfr4* REV | CTCCACAAGGCATGTGTATGT |  |
| *Kazald1* FOR | GTCCACAGGCCCCGATTAC | 212 |
| *Kazald1* REV | CGCATCGCCCGTAGAAGTTAG |  |
| *Myof* FOR | ACCGCTTTCGGTGTGATCC | 127 |
| *Myof* REV | GCCAGTAATGGTTTGGTGTCTTC |  |
| *Nr4a1* FOR | TTGAGTTCGGCAAGCCTACC | 100 |
| *Nr4a1* REV | GTGTACCCGTCCATGAAGGTG |  |
| *Pyroxd2*-FOR | CAAGGAGTCACACTGCAGGA | 159 |
| *Pyroxd2*-REV | GGTGACAGGAGACTGGGTGT |  |
| *Rgs2* FOR | GAGAAAATGAAGCGGACACTCT | 197 |
| *Rgs2* REV | GCAGCCAGCCCATATTTACTG |  |
| *Rps8* FOR | GCATCTCTCGGGACAACTGG | 118 |
| *Rps8* REV | CGAGGGCCAATCTTCGTGTT |  |
| *Scarb1* FOR | TTTGGAGTGGTAGTAAAAAGGGC | 71 |
| *Scarb1* REV | TGACATCAGGGACTCAGAGTAG |  |
| *Slc15a1* FOR | CCGGCACACCCTTCTAGTG | 173 |
| *Slc15a1* REV | TGGCGTTGTGACTGGTGAC |  |
| *Slc16a3* FOR | TCACGGGTTTCTCCTACGC | 167 |
| *Slc16a3* REV | GCCAAAGCGGTTCACACAC |  |
| *Star* FOR | CAGAGGATTGGAAAAGACACGG | 158 |
| *Star* REV | GGCATCTCCCCAAAATGTGTG |  |
| *Stard5* FOR | GGAGGCCATCTGAGGAGTTTC | 91 |
| *Stard5* REV | AACTGGCTTTATGCAATCCCA |  |
| *Tbx2* FOR | CCGATGACTGCCGCTATAAGT | 123 |
| *Tbx2* REV | CCATCCACTGTTCCCCTGT |  |
| *Tbx3* FOR | GAACCTACCTGTTCCCGGAAA | 121 |
| *Tbx3* REV | CAATGCCCAATGTCTCGAAAAC |  |
