# Supplementary material for: RiboTag Analysis of Actively Translated mRNAs in Sertoli and Leydig Cells In Vivo
Source: PLoS One. 2013 Jun 11;8(6):e66179. doi: 10.1371/journal.pone.0066179 (PMC3679032; doi:10.1371/journal.pone.0066179)
Supplement: Text S1 — Supplemental experimental procedures. (DOCX) [file pone.0066179.s015.docx]

**SUPPLEMENTARY EXPERIMENTAL PROCEDURES**

***Mice and treatments***

To determine the regulation of polyribosome-associated transcripts by testosterone (T) in Sertoli cells, adult AMH-cre: RiboTag mice (n=3) were injected with either saline, cetrorelix or cetrorelix + T. Mice were sacrificed after 4 h of T administration and RiboTag analysis was performed at this time point. Cetrorelix (50ug, Bachem) was given s.c. for 3 d before a single i.p. injection of 10 mg of testosterone enanthate (Delatestryl, Savient Pharmaceuticals). To assess the regulation of polyribosome-associated transcripts by FSH in Sertoli cells, a similar approach was performed using acyline as a GnRH antagonist and highly purified FSH instead of T in adult AMH-Cre: RiboTag mice. In this case, the response to FSH was assessed after 1 h and 4 h of FSH administration. Acyline (300 ug) was given s.c. for 3 d before a single i.p. injection of 1 unit of highly purified human FSH (>99.5%, Scripps laboratories). In both experiments, microarray analysis was performed on animals with confirmed T and FSH serum levels (n=2). Serum was obtained when the animals were sacrificed by cardiac puncture.

***Cluster analysis***

Transcripts showing significant differences between groups (p<0.01 using One-way Analysis of Variance (ANOVA)) were grouped according to their response to the treatments using dChip (http://biosun1.harvard.edu/~cli/complab/dchip/).
